# HIV-1 Epidemiology, Genetic Diversity, and Primary Drug Resistance in the Tyumen Oblast, Russia

**DOI:** 10.1155/2016/2496280

**Published:** 2016-11-13

**Authors:** Natalya M. Gashnikova, Ekaterina M. Astakhova, Mariya P. Gashnikova, Evgeniy F. Bocharov, Svetlana V. Petrova, Olga A. Pun'ko, Alexander V. Popkov, Aleksey V. Totmenin

**Affiliations:** ^1^State Research Center of Virology and Biotechnology Vector, Koltsovo, Novosibirsk Oblast 630559, Russia; ^2^Tyumen Regional Center for Prevention and Control of AIDS and Infectious Diseases, Ul. Novaya 2, Tyumen, Russia

## Abstract

*Introduction*. Specific molecular epidemic features of HIV infection in Tyumen Oblast (TO), Russia, were studied.* Methods*. The genome sequences encoding HIV-1 protease-reverse transcriptase, integrase, and major envelope protein were examined for 72 HIV-1 specimens isolated from the TO resident infected in 2000–2015.* Results*. The recorded prevalence of HIV-1 subtype A (A1) is 93.1%; HIV-1 subtype B continues to circulate in MSM risk group (1.4%). Solitary instances of HIV-1 recombinant forms, CRF63_02A1 (1.4%) and CRF03_AB (1.4%), were detected as well as two cases of HIV-1 URF63_A1 (2.8%). Phylogenetic analysis showed no HIV-1 clustering according to the duration of infection and risk groups but revealed different epidemic networks confirming that HIV infection spread within local epidemic foci. A high incidence of CXCR4-tropic HIV-1 variants and a higher rate of secondary mutations influencing the virus fitness (K20R, L10V, and I) are observed among the virus specimens isolated from newly infected individuals.* Conclusions*. The current HIV-1 epidemic in TO develops within the local epidemic networks. Similar to the previous period, HIV-1 subtype A is predominant in TO with sporadic cases of importation of HIV-1 recombinant forms circulating in adjacent areas.

## 1. Introduction

The Russian Federation is now in the list of regions with a high rate of HIV epidemic spread [1]. In 2016, the total number of officially registered persons with diagnosed HIV infection in Russia exceeds one million. The HIV prevalence among Russian citizens in 2015 continued to grow. A high HIV prevalence (over 0.5% of total population) is observed in 26 regions, housing 41.5% of the population of this country [2].

**Figure 1:**
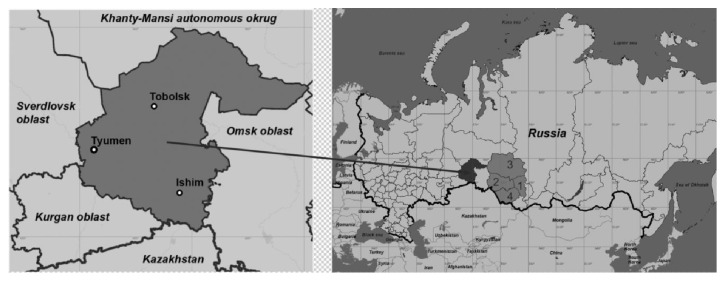
Geographical location of the Tyumen Oblast (highlighted in dark gray) and neighboring territories, Russia. (1) Kemerovo Oblast; (2) Novosibirsk Oblast; (3) Tomsk Oblast; and (4) Altai Krai.

The cases of first mass detection of HIV-1 infection in the territories of Russia and other newly independent states of the former Soviet Union date back to 1996. In 2000-2001, local dramatic outbreaks of HIV-1 infection were recorded in many regions of Russia; these outbreaks stemmed from an active spread of HIV-1 among injection drug users (IDUs). HIV-1 subtype A was included into the risk group for IDUs and continued its spread in many territories of this country [3–8]. The Tyumen Oblast (TO) is a federal subject of Russia with the administrative center in the city of Tyumen (Figure 1). This territory with an area of 1435 200 km^2^ has 3615 485 residents, with the urban population accounting for 80.12%. TO was among the first Russian territories actively involved in the HIV epidemic: 767 individuals diagnosed with HIV infection were at once recorded there in 1999 and 1067 individuals in 2000. A drastic increase in the HIV prevalence rate in TO in 1999–2002 was associated with a spread of opioid drugs (fresh opium and heroin), so that the infection cases associated with drug abuse accounted for 98–78% (98.4% in 1999, 96.4% in 2000, 93.7% in 2001, and 78.4% in 2002). The maximal number of cases was recorded in the city of Tyumen and neighboring areas: the share of HIV-infected individuals living in the city of Tyumen in 2000 amounted to 73.6% of the total detected cases.

**Figure 2:**
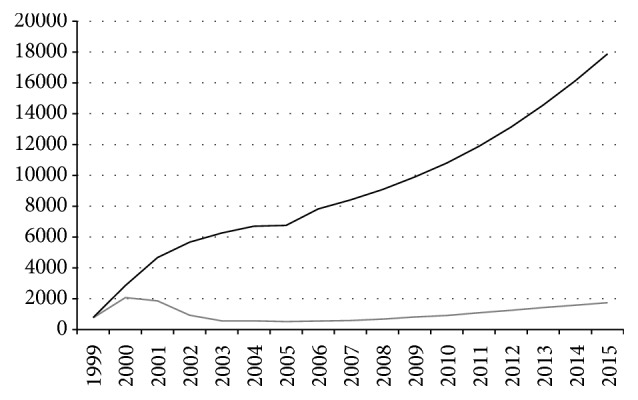
Dynamics of the HIV-1 epidemic in the Tyumen Oblast. Gray line denotes the newly diagnosed infection cases and black line total number of HIV-infected individuals.

Since the very beginning of mass HIV spread in TO, several different programs were implemented there that focused on HIV prevention and treatment (information and education of various population cohorts and organization of laboratory facilities allowing for timely detection, prescription, and efficiently control of the relevant therapies) and on the control of drug abuse. This allowed for coping with the drastic growth in the epidemic: its rate in 2003–2008 considerably slowed down (Figure 2). During this period, the minimal growth in HIV morbidity was observed (below 2% or even negative); however, the share of women among the newly diagnosed HIV infection cases increased as well as the contribution of sexual transmission (50% in 2004), while the share of teenagers among HIV-infected individuals reduced. During the overall epidemic, sexual transmission was to a larger degree characteristic of women: up to 70% are annually infected via heterosexual contacts and only 22–27% via drug versus 73–78% of men infected when using drugs and only 29–33%, via sexual contacts.

Since 2008, numerous new drugs and homemade drug variants have appeared in the drug market. Emergence of new psychoactive substances offered as a “legal” alternative for the known drugs [9] controlled at an international level had a significant effect on the further development of HIV epidemic in many regions of this country. The new psychoactive substances (the so-called “synthetics” and “salts”) are marketed under harmless names as “salts,” such as, bath salts, legal highs, speed euphoria, Barnyi, Cico, Cristalius SEX, Cristalius LOVE, Miff, Ivory Wave, Liquid Snow Berry, Snow Blow, and Spice. These substances were sold almost legally and were considerably less expensive as compared with classical drugs, making them easily accessible.

Synthetic psychoactive substances differ from the opioid drugs not only by their price and accessibility. These substances have a lower toxic threshold, which decreases the fear of death caused by overdosing, and have no effect of withdrawal; that is, they are used not to eliminate the withdrawal symptoms, but to reach an altered state of consciousness (euphoria or almightiness). Specific features of synthetics consist not only in their effect on the nervous system (disturbance of locomotion, memory, and sensitivity to pain as well as an increase in sexual sensations), but also in the injury of the immune system. The dependence on synthetics develops very rapidly. Ever new synthetics with changed structures and different administration methods (injection, smoking mixtures, etc.) constantly appear on the marker with addition of such substances to the lists of illegal drugs [10].

Emergence of such psychoactive substances involved many new persons into practicing risk behavior. The individuals intravenously injecting synthetics lose control over their behavior and start to share syringes. The practice of mindless unprotected sex becomes common when using some synthetics, including smoking mixtures. Consequently, the number of newly diagnosed HIV cases started to increase in TO in the second half of 2009. The epidemic history of HIV^+^ TO residents ever more frequently contains the use of synthetic psychostimulants.

Characteristic of the subsequent years is a more active aggressive spread of psychoactive substances in many Russian regions. It is possible to purchase synthetics just calling by phone or via the internet. The situation continued to deteriorate because of an increased accessibility of these synthetic psychostimulants and spread of the drugs effective in sexual disinhibition; in addition, many drug addicts commenced practicing combined (multiple) drug use. For 2011–2014, men were prevalent among the newly detected HIV^+^ individuals, also suggesting activation of the drug-associated HIV infection route. Nonetheless, a stable decrease in the annual rate of HIV morbidity growth has been observed starting from 2011 on the background of an absolute increase in the HIV incidence.

As of May 01, 2016, 18 423 cases of HIV infection were recorded in TO. According to the HIV prevalence, TO is the eighth in the list of Russian regions (1107.0 HIV^+^ individuals per 100 000 population) and thirteenth according to the morbidity rate (106.6 per 100 000 population).

Active HIV-1 outbreaks in the regions adjacent to TO (Novosibirsk, Tomsk, and Kemerovo Oblasts; Figure 1) recorded in 2008–2014 were associated with a drastic change in genetic characteristics of the circulating viruses, namely, replacement of the earlier circulating HIV-1 subtype A by CRF63_02A1 [11–13]. The studies of the HIV-1 variants circulating in TO have not been conducted so far. A high HIV-1 prevalence in TO and a long history of antiretroviral therapy (ART) there justify the interest to assess the specific molecular genetic features of the virus population circulating there and analyze HIV-1 primary resistance.

## 2. Materials and Methods

In total, 74 TO residents with diagnosed HIV infection were involved in this study. Peripheral blood of the HIV-infected individuals was sampled by medical staff of the Tyumen Regional Center for the Prevention and Control of AIDS and Infectious Diseases from September to December 2015 with pretest and posttest psychological consultations; epidemiologists obtained the written informed content from the participants and questioned them. The study of clinical blood samples complied with the ethical standards of the Helsinki Declaration of 1975 (http://www.wma.net/en/30publications/10policies/b3/), revised in 2008. The study projects were approved by the local ethical committee (protocols nos. 7 and 15 of March 20, 2013, and March 4, 2015). The blood samples were linked with demographic and clinical data via coded ID numbers according to the requirements of medical ethics in Russia. All participants were ART-naïve before the tests. The recorded characteristics for patients included their gender, age, transmission route, estimated time of infection, dates of the last negative and first positive tests for HIV, drug use, viral load, and CD4 cell count at the moment of diagnosis. The group of individuals HIV-infected when using drugs included those infected during nonmedical drug injection of drugs and psychoactive substances.

Virus RNA was isolated from 200 *μ*L of blood plasma using RealBest DeltaMag kits according to the manufacturer's protocol (Vector-Best, Russia). Amplification was performed using a lyophilized ready-to-use Reverse Transcription Master Mix containing all the components for a single-tube reverse transcription and PCR (Vector-Best, Russia) and an in-house set of primers. This allowed for amplifying three HIV-1 specific fragments for each clinical sample, namely, two fragments of the* pol* gene encoding the protease-reverse transcriptase (PR-RT, 1400 nt), integrase (IN, 960 nt), and a fragment encoding a part of the major envelope protein,* env* (732 nt). Sequencing was conducted in a 3130xl (Applied Biosystems, United States) automated sequencer. All original sequence fragments of the* pol* and* env* gene regions were assembled in whole sequences in Sequencher 4.1 software (Gene Codes Corporation, Ann Arbor, MI, United States). The assembled sequences of* pol* fragments (PR-RT and IN) and* env* gene fragments were compared to the corresponding reference sequences of various HIV-1 subtypes and recombinant forms extracted from the Los Alamos HIV-1 database using CLUSTALW Multiple Alignment and BioEdit software 7.2.5 [14]. Phylogenetic analysis was performed with MEGA 6.0.6 by neighbor-joining method with 1000 bootstrap replicates based on Kimura's two-parameter model [15]. Statistical significance of phylogenetic tree topologies was estimated using bootstrap analysis. New intersubtype or inter-CRF sequences were analyzed by recombination identification programs (RIP, https://www.hiv.lanl.gov/) and SimPlot 3.5.1 software using a 200 nt window with tree construction by the neighbor-joining method applying Kimura's two-parameter substitution model. The possible intersubtype mosaicisms of URFs were screened using the online jpHMM program (http://jphmm.gobics.de/submission_hiv.html) [16]. The V3 loop sequences were analyzed to estimate the genotypic virus tropism and to confirm the phenotypic coreceptor specificity using the online tools Position-Specific Scoring Matrix (PSSM) (http://indra.mullins.microbiol.washington.edu/webpssm/) and Geno2pheno [coreceptor] 2.5 (g2p) (http://coreceptor.bioinf.mpi-inf.mpg.de/index.php), additionally checking whether the sequence codes for positively charged amino acid residues at 11 and/or 25 codons of the V3 loop [17]. The analyzed* pol* gene sequences were assayed for the presence of mutations determining resistance to protease, reverse transcriptase, and integrase inhibitors (DR mutations) with the specialized online service (http://sierra2.stanford.edu/sierra/). The transmitted DR mutations were determined based on the WHO-recommended list of mutations for DR surveillance [18].

The HIV-1 polymerase, integrase, and* env *sequence data were submitted to the GenBank under the accession numbers KX640117–KX640184, KX640185–KX640251, and KX640252–KX640297.

## 3. Results

### 3.1. Clinical Epidemiological Data on Patients

In order to assess the current molecular epidemic situation on HIV-1 spread, 74 individuals diagnosed with HIV were involved in this study.  Table 1 lists the clinical epidemiological data for the examined patients. The HIV-specific fragments of necessary lengths were not obtained for two HIV-1 samples, so they were discarded. Correspondingly, the examined sample comprised 72 individuals, namely, 33 (45.8%) men and 39 (54.2%) women, with an average age of 35 years (range, 23–60); 33 persons reported injection drug use (IDU); five of them were sex workers; 41 individuals (56.9%) were infected via heterosexual contacts; three of them were IDUs; and one man was an MSM (1.4%). Among the IDUs, 27.3% admitted intravenous use of heroin and/or fresh opium; 42.2% used salts and/or synthetic psychostimulants along with heroin/fresh opium; 27.3% used synthetic psychostimulants; and one participant refused to answer this question. Of them, 82.1% were residents of the city of Tyumen and 18.1% lived in other areas of the oblast. One person (no. 23) was putatively infected beyond the Tyumen Oblast and the remaining participants were infected in Tyumen/Tyumen Oblast. Putative time of infection was reliably enough detectable for 58.3% of the participants, since they had been earlier examined for HIV. In particular, 13.9% of the participants had been infected before 2010 and 44.4%, presumably in 2010–2015. According to analysis of the clinical data, the median viral load and CD4^+^ cell counts were 2.2 × 10^4^ (5.0 × 10^2^–7.0 × 10^6^) copies/mL and 332 (2–1240) cells/*μ*L for the overall sample and 2.2 × 10^4^ (5.0 × 10^2^–3.4 × 10^5^) copies/mL and 450 (46–1105) cells/*μ*L for the individuals infected in 2013–2015, respectively. Analysis of epidemiological data of the patients involved in the study demonstrated that this sample contained the clinical specimens of the individuals infected at different periods of the epidemic, including at least 44% of the specimens of the persons infected with HIV-1 after 2010. Examination of the genetic characteristics of HIV-1 variants isolated from the collected clinical specimens allows for molecular genetic description of the specific features in HIV infection epidemic and of the virus variants that determine the current epidemic in TO.

### 3.2. Analysis of Genetic Heterogeneity of the Virus Variants Isolated from HIV-Infected Individuals

**Figure 3:**
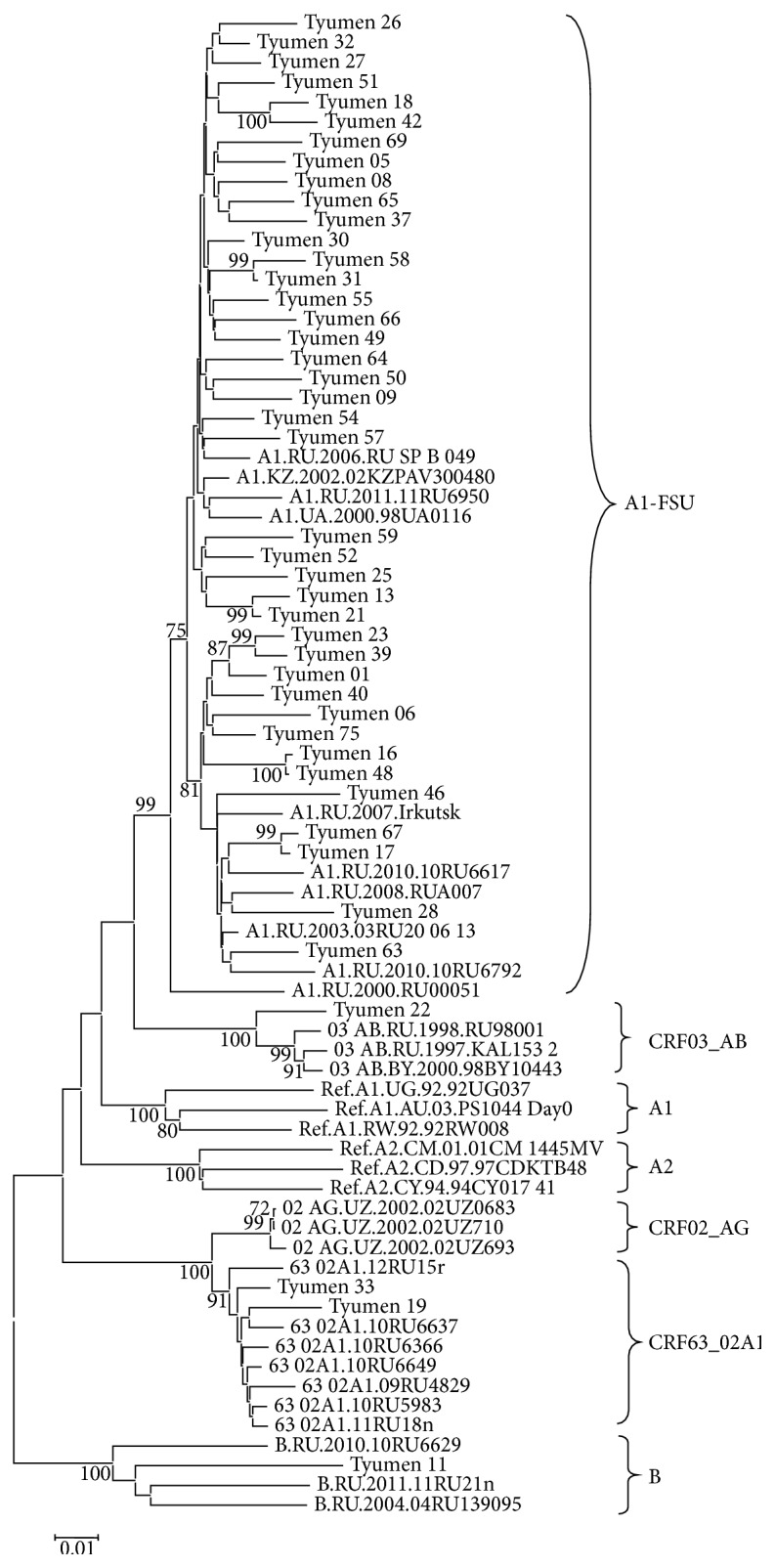
Neighbor-joining phylogenetic tree analysis of HIV-1 pol gene fragment (PR-RT) sequences from HIV-infected residents of Tyumen Oblast. Genetic distances were estimated using the Kimura's two-parameter model; clustering of strains was tested with 1000 bootstrap replicates; and statistical significance of the phylogenetic tree topology was estimated using bootstrap analysis.

**Figure 4:**
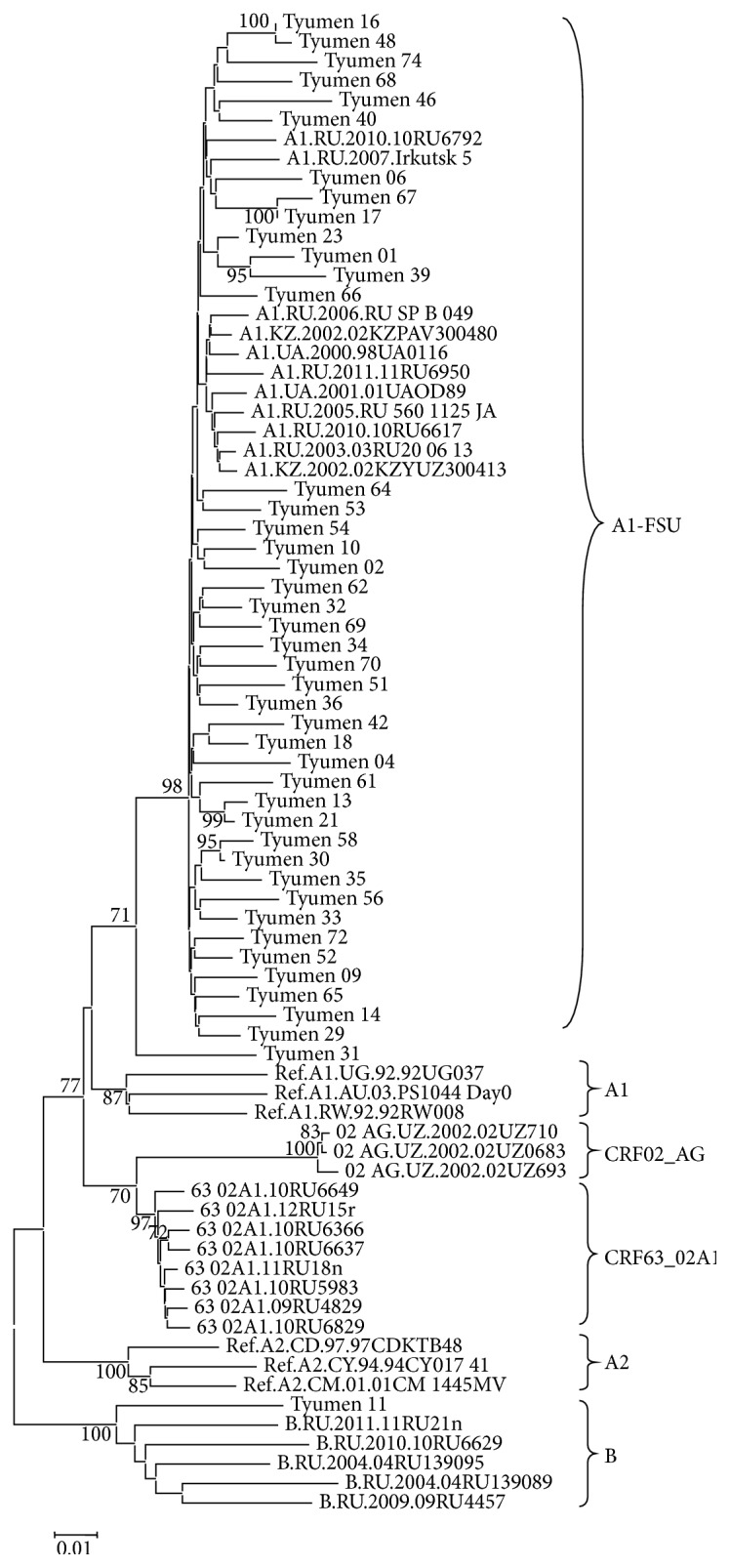
Neighbor-joining phylogenetic tree analysis of HIV-1* pol *gene fragment (IN) sequences from HIV-infected residents of Tyumen Oblast. Genetic distances were estimated using Kimura's two-parameter model; clustering of strains was tested with 1000 bootstrap replicates; and statistical significance of the phylogenetic tree topology was estimated using bootstrap analysis.

The virus DNA was isolated from the blood plasma samples of 72 individuals, and the fragments encoding HIV-1 PR-RT (70 samples), IN (68 samples), and envelope protein (50 samples) were amplified and subject to phylogenetic analysis individually for each genome region. The phylogenetic trees constructed for the* pol* gene nucleotide sequences (PR-RT and IN) are shown in Figures 3 and 4. The genotyping of virus variants according to the PR-RT region demonstrates that one HIV-1 variant (Tyumen 11) clustered with the HIV-1 subtype B; one (Tyumen 22) with CRF03_AB; and two (Tyumen 19 and Tyumen 33) with CRF63_02A1; the remaining assayed virus variants clustered with HIV-1 subtype A (A1). The following distribution of the analyzed virus specimens was observed for the HIV-1 IN region (Figure 4): Tyumen 11 clustered with subtype B; Tyumen 31 belongs to subtype A according to PR–RT and is intermediate between subtype A and CRF63 02A1 according to IN region, while Tyumen 33 (genotyped as CRF63 02A1 according to PR-RT) together with most HIV-1 variants clustered with subtype A.

**Figure 5:**
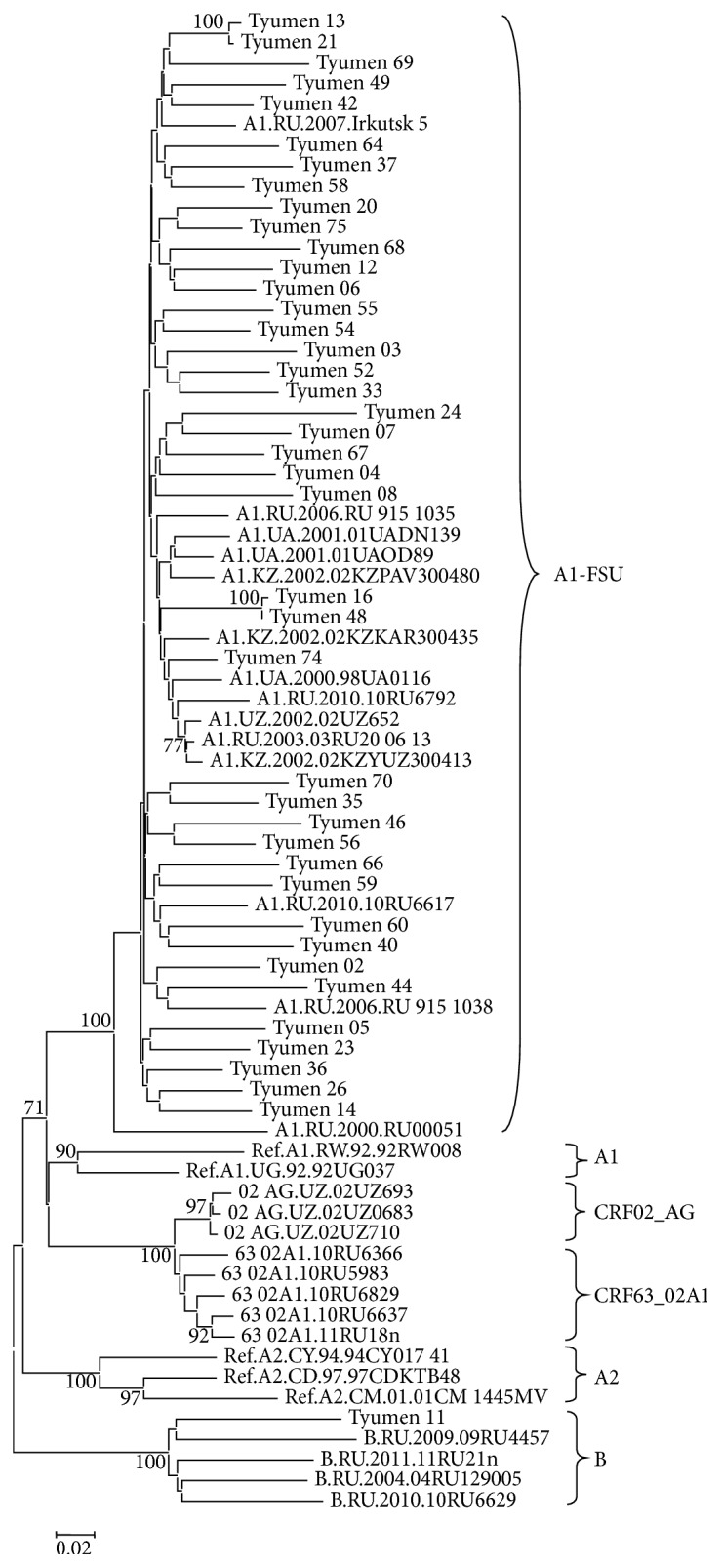
Neighbor-joining phylogenetic tree analysis of HIV-1* env *gene fragment sequences from HIV-infected residents of Tyumen Oblast. Genetic distances were estimated using Kimura's two-parameter model; clustering of strains was tested with 1000 bootstrap replicates; and statistical significance of the phylogenetic tree topology was estimated using bootstrap analysis.

The analysis of* env* gene region confirmed the Tyumen 11 clustering with subtype B, while Tyumen 31 and Tyumen 33 were genotyped as HIV-1 subtype A (Figure 5).

The conducted phylogenetic analysis revealed several epidemic networks in HIV-1 distribution. In particular, six pairs of genetically related HIV-1 variants were discovered; these variants clustered together in the phylogenetic tree constructed based on HIV-1 PR-RT sequences with bootstrap values of ≥99%. Interestingly, these pairs unite the virus variants isolated from the individuals infected in different years (Tyumen 13 and Tyumen 21 and Tyumen 31 and Tyumen 58). For three of them, the branch topologies are also confirmed by the phylogenetic analysis based on the IN and/or* env* sequences (Tyumen 17–Tyumen 67, Tyumen 13–Tyumen 21, and Tyumen 16–Tyumen 48), thereby suggesting a high probability of the epidemic relation between these pairs of patients.

As for the pair Tyumen 18–Tyumen 42 and Tyumen 23–Tyumen 39, their support for sharing the same subbranch according to IN region was statistically insignificant (bootstrap values of <70%), while Tyumen 31 and Tyumen 58 variants belong to different HIV-1 genovariants (Figures 3 and 4). The obtained data suggest reinfection of the patients Tyumen 31, Tyumen 18, and/or Tyumen 42 and Tyumen 23 and/or Tyumen 39.

Summing up the genotyping results, we conclude that HIV-1 subtype A still remains predominant and determines development of the current epidemic in TO, its prevalence being 93.1%. HIV-1 subtype B continues its circulation in MSM risk group and was detected in one case (1.4%). The only cases of HIV-1 recombinant forms CRF63_02A1 (1.4%) and CRF03_AB (1.4%) were detected as well as two instances (Tyumen 31 and Tyumen 33) of HIV-1 unique recombinant forms, URF63_A1. The URF genome is mosaic, partially identical to subtype A and partially to CRF63_02A1, as was confirmed by phylogenetic analysis: some studied loci of the same isolate belong to subtype A1 and the others to subtype 63_02A1 (Figures 3 and 4).

### 3.3. Analysis of Primary Resistance and Tropism of the Studied HIV-1 Variants

All HIV-1 nucleotide sequences encoding protease (*n* = 70), reverse transcriptase (*n* = 70), and integrase (*n* = 68) of the isolated virus variants were assayed for the presence of mutations influencing the resistance to the corresponding virus reproduction inhibitors, namely, the inhibitors of protease (PIs), reverse transcriptase (nonnucleoside and nucleoside inhibitors, NNRTIs, and NRTIs, resp.), and integrase (IIs) (Table 2).

The conducted analysis demonstrated a low prevalence of the primary drug resistance (DR) among the studied variants of HIV-1. Nonpolymorphic mutations determining HIV-1 resistance to NNRTIs, G190E, and Y181C were detected only in two HIV-1 specimens circulating in TO. The former mutation causes a high-level resistance to NVP, EFV, RPV, and ETR, while the latter is associated with a decreased response to the therapy with EFV. Likewise, two cases of the resistance to IIs were found. The corresponding mutations are T66I and E157Q. The former is a major mutation selected in the individuals treated with EVG, decreasing 15-fold the sensitivity to the drug, and the latter is a secondary mutation, reducing fivefold the sensitivity to RAL and halving the sensitivity to EVG.

The polymorphic mutations associated with drug resistance had different prevalence. The mutations able to improve HIV-1 reproductive characteristics in the presence of PIs or decrease the PI sensitivity (K20R, T74S, L10V/I, and K20I) were most frequently recordable among the examined HIV-1 variants, mainly in the individuals infected in 2010–2015. Solitary cases of the mutations influencing HIV resistance to PIs in combination with other DR mutations (L33F and A71I) were also recorded.

Of the mutations influencing NRTI therapy, only the V118I mutation (of the TAM set, efficient only in combination with other resistance mutations) was found. The mutations with a low effect on the resistance to NNRTIs (E138A, V108I, and V90I) also had a low prevalence. The sequences encoding HIV-1 IN carried the L74I in 94.1% of the examined subtype A viruses. This allows L74I to be regarded as a characteristic mutation for the HIV-1 subtype A population circulating in this area. On the other hand, only the A62VRT mutation of the DR mutations in the pol gene specific of HIV-1 subtype IDUA (V77I_PR_ and A62V_RT_) was detected at a rate of 8.3%. The mutation A62V is an accessory mutation that often occurs in combination with the multinucleoside resistance mutations K65R or Q151M. Alone it does not reduce NRTI susceptibility.

The tropism of examined HIV-1 variants, that is, the use of CCR5/CXCR4 coreceptors, was predicted by analyzing the deduced amino acid sequences of HIV-1* env* gene V3 loop. The prevalence of CXCR4-tropic variants among the examined HIV-1 specimens was 20%; these variants were isolated from the individuals infected over 5 years ago in 25% of the cases versus 13.6% of the individuals infected less than 3 years ago.

## 4. Discussion

The circulation of resistant strains in the population of people living with HIV/AIDS (PLHIV) is a serious factor influencing the selection of first-line antivirals and adoption of the strategies for efficient ART schemes. Unlike the HIV IIs, which in this country are used only recently, the PIs and RTIs were and are still widely used for the specific ART. In Russia and the CIS countries, the HIV variants carrying DR mutations in* pol* gene are most frequently detectable in the individuals with inefficient ART. Among them, the mutations G190S, K103N, M184V, Y181C, K101E, M41L, and T215F/Y, influencing the HIV resistance to NRTIs and NNRTIs, are observed at a high rate [19–24]. Mutations K103N, M184I, T215Y, G190S, Y181C, K65R, and V108I with prevalences of 0.3 to 7% depending on the region are most frequently detected in the naïve HIV-infected individuals [21–28]. Only two HIV-1 variants carrying solitary G190E and Y181C mutations (causing resistance to RTI) and two HIV-1 specimens with resistance to IIs were found in the examined sample of HIV-1 specimens. Detection of the mutations associated with the resistance to IIs on the background of a very limited use of these drugs in clinical practice is explainable by a random selection of such substitutions. Despite a long-term practice of treatment with NRTIs, NNRTIs, and PIs, the transmission of resistant virus variants in the HIV population isolated from infected ART-naïve residents in TO was low. A low prevalence of the HIV-1 primary DR may be a favorable prognostic factor in the epidemic development in TO. However, note that over 10% of the HIV-1 variants circulating in TO had the mutations potentially associated with a decreased sensitivity to PIs and improvement of HIV reproductive characteristics in the presence of PIs.

The mutations A62V_RT_ and V77I_PR_ are certain marker mutations for the subtype A HIV-1 variants circulating in Russia. The circulating subtype A HIV-1 variants characteristic of Russia are those carrying the V77I_PR_ and/or A62V_RT_ mutations and wild-type (WT) HIV-1. The prevalence of such viruses among both the naïve HIV-infected individuals and the patients undergoing ARV therapy considerably varies ranging from solitary cases to 80–100% [27–29]. Presumably, this is associated with the importation history of the different subtype A HIV-1 variants to certain regions of the country and with the specific features in subsequent progression of the epidemic. Wild-type HIV-1 viruses were detected in TO as early as the late 1990s [30]. The observed low abundances of A62V_RT_ (8.3%) and V77I_PR_ (11.4%) among the HIV-1 variants in TO may result from importation of WT HIV-1 to this area followed by prevalent development of the “own” focus of HIV-infection epidemic.

The use of CCR5 antagonists in the modern therapeutic schemes makes it necessary to study the tropism of the HIV-1 variants isolated from the ART-naïve patients in order to assess the potential of such drugs [31–33]. According to several studies, the onset of HIV-1 infection with the spread of CCR5-tropic viruses in the body is characteristic of HIV-1 subtype A as well as most virus genetic variants except for subtype D. Different studies on the use of CCR5 or CXCR4 coreceptors by HIV-1 belonging to non-B subtypes (including subtype A) involving naïve HIV^+^ patients demonstrate that the abundance of CXCR4-tropic viruses varies from 2 to 40%. This high variability may be influenced by different compositions of the studied populations and by differences in methodology. According to the data of most researchers, the detection rate of CXCR4-tropic HIV-1 variants in the patients at early disease state amounts to only several percent. Typically, the abundance of CCR5-tropic HIV variants decreases with the disease progression so that the CXCR4-tropic viruses emerge only at later HIV infection stages [34–43].

The results of our work demonstrate that the number of HIV-1 variants using CXCR4 coreceptor also increases with the duration of the disease among the studied subtype A viruses isolated from the patients with confirmed date of infection: the CXCR4-tropic viruses were isolated from 13.6% of the subjects infected several months to 3 years ago and 25% of the subjects infected over 5 years ago, which agrees with the data of other researchers. Nonetheless, the recorded levels of CXCR4-tropic HIV-1 variants among the patients infected for less than 3 years are rather high. Our results demonstrate that it is necessary to study the tropism of a virus before planning the use of CCR5 antagonists in the therapeutic schemes for the individuals infected with HIV-1 subtype A and to additionally study the prevalence of CXCR4-tropic HIV-1 variants among the naïve patients infected with other genetic variants specific of different regions of this country.

Comparison of the HIV-1 genetic variants isolated from the infected TO residents in the 2000s (2000–2009) and later (2010–2015) did not reveal any significant differences in the circulating virus variants. HIV-1 subtype A was and still remains the predominant variant. Phylogenetic analysis did not find any clustering of HIV-1 subtype A according to the time of infection and risk groups.

Different epidemic networks revealed by phylogenetic analysis of the HIV-1 variants, on the one hand, confirm that HIV infection spreads within local epidemic foci formed in TO during the previous years. On the other hand, the existence of such epidemic chains that include the HIV-1 variants with a high degree of genetic identity should be taken into account in a correct epidemiological investigation. Genetic identities of HIV-1 variants should be obligatorily assessed according to several genome regions. The revealed epidemic networks, which suggest that HIV is spread within an established focus of HIV infection, are also confirmed by analysis of the epidemic progression.

After 16 years of the epidemic in TO, the age cohort of 30–39 years has emerged to be maximally affected. The share of this age cohort is constantly growing, from 6.9% in 2000 to 48–50% in 2014-2015. The share of the individuals over 50 years old is also constantly growing (0.8% in 2000 to 4.6% in 2014). Presumably, part of the newly diagnosed patients has long been infected and, possibly, some “former” IDUs returned to using drugs and were thus involved into the epidemic. Epidemiological investigations recently performed when newly diagnosing a HIV infection demonstrates that HIV reinfection in the local foci of territorial epidemic remains rather high. The cause in the cohort free of drug addiction is a low sexual culture; as for the IDUs, especially using psychostimulants, they almost or completely neglect any of protection tools. Presumably, all of these factors also enhance HIV reinfection.

The detected HIV-1 URF63_A1, the so-called secondary recombinant forms, could have emerged only as a result of reinfection by different HIV-1 genetic variants (subtype A and CRF63_02A1). In the examined group, one patient (Tyumen 31) infected by URF63_A1 practiced intravenous heroin use in the early 2000s and then had accidental unprotected sexual contacts and another patient (Tyumen 33) intravenously uses synthetic psychostimulants since 2012, having a drug-associated contact with a HIV^+^ partner and accidental unprotected sexual contacts in his history. They have never left the TO area, suggesting that distinct HIV-1 URF63_A1 genetic variants circulate in TO. A case of isolation of an ancestral HIV-1 variant, CRF63_02A1, of a TO resident who is a long-term drug user confirms the possibility that new URFs emerge in TO. Presumably, the HIV-1 CRF63_02A1 originated from adjacent Siberian territories (Novosibirsk, Tomsk, and Kemerovo Oblasts; Figure 1), where this genetic variant is actively spreading in recent years [11–13].

Despite that the territorial epidemics in the Siberian region and TO display similar characteristics (distribution of HIV-infected individuals according to infection routes, social parameters, gender, age, spread of drug abuse, and types of drugs), the epidemic in Siberia started with a delay of several years. Unlike Novosibirsk, Tomsk, and Kemerovo Oblasts, where the epidemic outbreaks with severalfold increase in the number of newly infected individuals were recorded in 2008–2014 (these territories are still at the top of the Russian rating for increase in newly infected persons), the last wave of HIV spread activation in TO was recorded in 2008–2011. Moreover, the epidemic growth rate was significantly lower: the maximal values recorded in 2009–2011 did not reach 20%; moreover, a slow but stable trend of a decrease in the HIV spread is observed after 2011. Presumably, a likely reason underlying such a “controlled” epidemic in TO is its early onset and, as a consequence, timely development of the medical facilities able to constantly provide preventive activities, professional pretest and posttest services for HIV-infected individuals, and psychological consulting as well as maintenance of the patients from diagnosing and registration to observation and timely therapy. Note that the therapy coverage in TO in 2015 was 98% and its efficiency according to the approved criteria was 79.6% [44].

Despite an apparent positive dynamic of this epidemic, the number of HIV-infected individuals in TO continues to grow. Currently, TO is the region attractive for migrant workers from both the CIS countries and other Siberian regions, where the current epidemics are determined by HIV-1 recombinant forms, CRF02_AG and CRF63_02A1. Therefore, these HIV-1 genetic variants can be imported to TO and spread there, demonstrating the importance of monitoring of the spreading HIV-1 variants on a regular basis, especially, in remote areas with constant presence of seasonal migrant workers.

## Figures and Tables

**Table 1:** Epidemic and clinical characteristics of HIV-infected individuals involved in the study and genotyping data on the isolated HIV-1 variants.

Number	^a^Gender	Age, years	^b^Date last tested negative	^c^Date first tested positive	^d^Assumed time of infection	^e^Transmission risk group	^f^Types of drugs	Plasma HIV-1 RNA copies/mL	CD4^+^ T cell count, cells/mm^3^	^g^HIV-1 tropism	HIV-1 genotype
1	M	38	2005	14.02.2007	2005–2007	CSW	O, H	7490	394	CCR5	A
2	F	37	^h^ND	14.09.2011	2010-2011	CSW	Sy	20 200	332	CCR5	A
3	F	27	2013	17.09.2015	2013–2015	HTs	—	4050	296	CCR5	A
4	F	31	ND	22.09.2015	2011–2013	HTs	—	1340	599	CCR5	A
5	M	36	2012	05.08.2014	2012–2014	IDU	O, H, Sy	132 000	199	CCR5	A
6	F	33	2003	24.09.2015	2003–2015	HTs	—	4020	428	CXCR4	A
7	M	39	2012	24.10.2012	2012	IDU	O, H, Sy	43 900	232	CCR5	A
8	M	37	ND	08.05.2001	2000	IDU	H	110 000	2	CXCR4	A
9	M	32	2010	29.09.2015	2011–2015	IDU	O, H, Sy	548	253	ССR5	A
10	F	35	2009	01.07.2015	2009–2015	IDU	H	15 900	210	^h^NA	A
11	M	38	ND	01.10.2015	2015	MSM	—	1060 000	284	CCR5	В
12	M	30	2015	24.09.2015	2014-2015	IDU	S	1080	413	CCR5	A
13	M	34	2015	09.10.2015	2015	HTs	—	36 100	1105	CCR5	A
14	F	36	2009	18.09.2015	2009–2015	HTs	—	2670	266	CCR5	A
15	F	40	2013	17.09.2015	2013–2015	HTs	—	43 700	332	NA	A
16	M	33	2006	16.10.2015	2007–2015	HTs	—	7100	285	CCR5	A
17	F	40	2015	02.10.2015	2014-2015	IDU	S, H, Sy	4490	353	NA	A
18	M	31	2009	06.10.2015	2009–2015	IDU	Sy	7150	243	NA	A
19	M	36	ND	19.03.2015	2001–2015	IDU	H, S	500	74	NA	CRF63_02A1
20	F	24	ND	29.09.2015	2010–2015	HTs	—	1120	500	CCR5	A
21	M	36	ND	15.11.2000	1999-2000	IDU	O, H	293 000	159	CCR5	A
22	M	34	2014	17.12.2014	2013-2014	HTs	—	5070	617	NA	CRF03_AB
23	F	28	ND	28.11.2011	2007–2010	IDU	H, S	217 000	398	CCR5	A
24	F	34	ND	15.10.2015	2013–2015	IDU	S	11 500	318	CCR5	A
25	F	34	2011	25.08.2015	2011–2015	HTs	—	500	666	NA	A
26	F	27	ND	15.10.2015	2012–2015	HTs	—	32 700	254	CCR5	A
27	F	30	2015	05.10.2015	2014-2015	IDU	S	22 960	641	NA	A
28	F	41	2013	19.10.2015	2013–2015	HTs	—	94 000	745	NA	A
29	M	39	ND	20.06.2002	2001	IDU	H	41 200	1240	NA	A
30	M	36	ND	29.04.2010	2010	IDU	H	239 000	250	NA	A
31	M	37	ND	20.12.2006	2002–2005	IDU	H	1150 000	90	CCR5	URF63_A1
32	F	34	ND	14.04.2014	2007–2010	HTs	—	11 400	296	NA	A
33	M	36	ND	23.07.2015	2012–2014	IDU	S	78 400	211	CCR5	URF63_A1
34	F	45	ND	02.10.2015	2007–2010	HTs	—	160 000	358	NA	A
35	F	36	2013	22.10.2015	2013-2014	IDU	H, S	4270	317	CCR5	A
36	M	45	2002	19.10.2015	2002–2015	IDU	O, H	88 400	208	CCR5	A
37	F	47	2013	11.11.2013	2012-2013	IDU	H, S	83 700	262	CXCR4	A
38	F	29	2007	26.10.2015	2007–2015	HTs	—	45 200	236	CXCR4	A
39	M	43	2013	09.10.2015	2013–2015	IDU	H, S	57900	494	CCR5	A
40	M	44	2009	01.10.2015	2009–2015	HTs	—	234 000	203	CCR5	A
41	M	26	ND	08.10.2013	2012-2013	IDU	S	9640	317	NA	А
42	F	35	2013	15.10.2015	2013–2015	HTs	—	24 100	859	CCR5	A
43	M	42	ND	23.07.2015	2008–2015	HTs	—	7040 000	114	CXCR4	A
44	F	31	2010	27.10.2015	2010–2015	HTs	—	60 400	452	CXCR4	A
45	F	25	2014	23.10.2015	2014-2015	HTs	—	20 200	376	NA	NA
46	M	36	2014	05.10.2015	2014-2015	HTs	—	344 000	470	CCR5	A
47	F	19	ND	23.10.2015	2013–2015	HTs	—	500	588	NA	NA
48	F	33	ND	01.10.2015	2014-2015	HTs	—	118 000	393	CCR5	A
49	F	31	ND	08.10.2015	2005–2015	HTs	—	110 000	290	CCR5	A
50	F	33	ND	19.10.2015	2010–2015	HTs	—	500	901	NA	А
51	F	36	ND	15.10.2015	2013-2014	HTs	—	10 500	1006	NA	A
52	M	34	ND	22.10.2015	2010–2015	HTs	—	138 000	197	CCR5	A
53	M	29	ND	19.10.2015	2003–2015	IDU	H, S, Sy	56 000	700	NA	A
54	F	47	ND	06.11.2015	2005–2015	HTs	—	15 800	589	CCR5	A
55	F	28	2013	17.09.2015	2013–2015	HTs	S	22 000	802	CCR5	A
56	M	37	ND	29.10.2015	2002–2015	HTs	—	225 000	434	CCR5	A
57	F	37	2008	23.10.2015	2010–2015	HTs	—	869	901	NA	A
58	F	33	ND	19.10.2015	2012–2015	HTs	—	12 100	205	CCR5	A
59	F	45	ND	21.08.2013	2012-2013	IDU	H, S	8720	193	CXCR4	A
60	F	32	2013	10.11.2015	2014-2015	IDU	S	500	281	CXCR4	A
61	M	35	ND	02.11.2015	2000–2005	IDU	H, S, Sy	33 800	181	NA	A
62	M	61	ND	11.11.2015	2010–2015	HTs	—	16200	206	NA	A
63	M	41	2013	02.11.2015	2013-2014	HTs	—	2210	379	CCR5	А
64	M	32	2013	06.10.2015	2014	IDU	H, S, Sy	41 800	450	CCR5	A
65	F	43	2012	06.11.2015	2012-2013	HTs	—	35 600	117	CXCR4	A
66	M	32	2010	09.11.2015	2015	IDU	H	671 000	277	CXCR4	A
67	F	37	2014	27.10.2015	2014-2015	HTs	—	18 400	411	CCR5	A
68	F	25	2014	20.11.2015	2014-2015	HTs	—	34 300	325	CCR5	A
69	F	37	2013	20.11.2015	2015	IDU	O, Sy, H, S	21 200	575	CCR5	A
70	F	37	2014	10.11.2015	2014-2015	HTs	—	8780	461	CCR5	A
72	M	39	2013	10.08.2015	2013–2015	HTs	—	283 000	46	CCR5	A
73	F	34	2014	24.07.2015	2014-2015	HTs	—	1110	942	NA	A
74	M	55	2013	06.11.2015	2013–2015	HTs	—	6810	707	CCR5	А
75	F	36	2003	30.11.2015	2014-2015	HTs	—	11 000	614	CCR5	А

^a^M: male and F: female. ^b^Data at the time of HIV-negative diagnosis. ^c^Data at the time of HIV-positive diagnosis.

^d^Assumed time of infection determined after individual interview with epidemiologist. ^e^Transmission risk groups; CSW: commercial sex worker; IDU: injection drug user; MSM: man having sex with man, and HTs: heterosexual contacts. ^f^Types of used drugs: H: heroin; O: other opioids; S: salt; and Sy: synthetic. ^g^Prediction using Geno2pheno 2.5 software and WebPSSM. ^h^ND: not detected; NA: not amplified.

**Table 2:** Detection rate and range of mutations in the *pol* gene in the examined HIV-1 variants that influence the sensitivity to virus reproduction inhibitors.

HIV-1 mutations associated with DR mutation (rate, %)			
Resistance to PIs, *n* = 70^*∗*^	Resistance to NRTIs, *n* = 70^*∗*^	Resistance to NNRTIs, *n* = 70	Resistance to IIs, *n* = 68
K20R (12.5), T74S (12.5), L10V.I (11.1), K20I (5.6), L33F (2.8), A71I (1.4)	A62V (8.3), V118I (6.9), T69S (4.2)	E138A (4.2), G190E (1.4), Y181C (1.4), N108I (1.4), V90I (1.4)	L74I (94.1), T66I (1.5), T97A (1.5), E157Q (1.5), T97A (1.5)

^*∗*^ *n*: the number of analyzed sequences.
